# Supplementary material for: Time to death from cervical cancer and predictors among cervical cancer patients in Felege Hiwot Comprehensive Specialized Hospital, North West Ethiopia: Facility-based retrospective follow-up study
Source: PLoS One. 2022 Jun 24;17(6):e0269576. doi: 10.1371/journal.pone.0269576 (PMC9232151; doi:10.1371/journal.pone.0269576)
Supplement: Attachment — Submitted filename: Response to reviewers.docx [file pone.0269576.s001.docx]

***Response to reviewers***

***Journal Requirements:***

1. Please ensure that your manuscript meets PLOS ONE's style requirements, including those for file naming.

*We thank you dear editor and we have made an effort that the manuscript meets PLOS ONE's style requirements.*

2. Please provide additional details regarding participant consent. In the ethics statement in the Methods and online submission information, please ensure that you have specified what type you obtained (for instance, written or verbal, and if verbal, how it was documented and witnessed). If your study included minors, state whether you obtained consent from parents or guardians. If the need for consent was waived by the ethics committee, please include this information.

*We admire your concern dear and we have provided the ethics statement in the Methods and online submission information clearly.*

3. Please ensure that you refer to Figure 1 in your text as, if accepted, production will need this reference to link the reader to the figure.

*Thank you we accept your comment and we referenced figure 1 in the main document.*

4. Please upload a copy of Figure 10, to which you refer in your text on pages 24 and 27. If the figure is no longer to be included as part of the submission please remove all reference to it within the text.

*We accept your comment and we have tried to make a correction on figures in general throughout the document.*

5. Please review your reference list to ensure that it is complete and correct. If you have cited papers that have been retracted, please include the rationale for doing so in the manuscript text, or remove these references and replace them with relevant current references. Any changes to the reference list should be mentioned in the rebuttal letter that accompanies your revised manuscript. If you need to cite a retracted article, indicate the article’s retracted status in the References list and also include a citation and full reference for the retraction notice.

*Thank you dear for your concern and we have revised all the references for their completeness and made necessary corrections.*

***Reviewer 1***

1. Title of the study not written clearly. Predictors of what?

*Thank your dear reviewer for your appreciable comment and we have made an amendment on the topic to make clear.*

2. First time abbreviation usage in the article not explained in a bracket but rather put at the end of the article.
   *Thank you dear and we have incorporated your concern in the main manuscript.*
3. In the abstract "being initiated with radiotherapy" is mentioned to increase the risk of death from cervical cancer, This is not clear to readers. Is the treatment really increasing the risk of death or is there another cofactor with those patients receiving radiotherapy? Otherwise this is technically/scientifically unsound.

*Thank you for your interesting comment and we have corrected the statement based on our finding in the main document.*

4. The conclusion drawn is not coherent with the data presented. The data did not presented about Treatment of comorbidities yet it is stated as "Treatment of comorbidities in the early stage of cervical cancer plays a key role in maximizing the survival time of cervical cancer patients."

*Thank you so much dear and we have revised the conclusion part that should be inconsistent with the finding.*

5. The Language used requires editing, "TARH" in the introduction section and "radiological treatment" in the discussion part are some of the words to mention.

*Great we thank you for your comment and we tried to edit all the document by language experts once again.*
